# Evaluation of the Role of C-reactive Protein as a Prognostic Indicator in Oral Pre-malignant and Malignant Lesions

**DOI:** 10.7759/cureus.60812

**Published:** 2024-05-21

**Authors:** Hamza Salema, Samir Joshi, Sudhir Pawar, Vivek S Nair, Vedangi V Deo, Manali M Sanghai

**Affiliations:** 1 Oral and Maxillofacial Surgery, Bharati Vidyapeeth Dental College and Hospital, Pune, IND

**Keywords:** prognosis, c-reactive protein, carcinoma, biomarkers, leukoplakia

## Abstract

Biopsy is the gold standard in the diagnosis of oral pre-malignant and malignant cases. In borderline cases, false-positive or false-negative results can grossly affect treatment planning, leading to a bad prognosis. C-reactive protein (CRP) has been linked to poorer outcomes for patients with oral pre-malignant and malignant lesions. To validate the histopathological finding and ultimately direct treatment, the study aims to correlate pre-treatment levels of CRP in oral pre-malignant and malignant lesions. This will provide a biomarker to assess the prognosis in such cases. Our study investigated 53 patients, out of whom 35 were males and 18 were females. A CRP analysis was performed on each patient. The automated immunoturbidimetric method was utilized to quantify CRP levels. The CRP values of pre-malignant lesions ranged from 2.46±1.79 mg/L, while the malignant group's levels ranged from 7.90±3.18 mg/L. The findings imply that plasma CRP levels may be a potential indicator of elevated cancer risk and that pre-diagnostic CRP concentrations are linked to the later development of oral cancer.

## Introduction

Oral cancer is one of the most common cancers globally, with a wide geographic range in incidence [1]. It is linked to the use of alcohol, betel nuts, tobacco, and other tobacco products. It results in high rates of morbidity and mortality, as well as financial, social, and familial difficulties.

Oral squamous cell carcinoma (OSCC) is the most prevalent kind of oral cancer, contributing to 80% to 90% of cases [2]. OSCC is the fourth most common cancer in India among those that affect women, after cancers of the breast, cervix, and ovary. The most common site of OSCC diagnosis in the Indian subcontinent is the buccal mucosa and gingiva, which is consistent with the use of chewable tobacco, which is frequently inserted into the buccal vestibule [3].

Numerous factors, including age, gender, time of diagnosis, socioeconomic status, pre-operative staging, tumor, node, and metastasis (TNM) classification, and histopathological features, influence the survival rate of patients with OSCC. Carcinogenesis causes cells to proliferate quickly, destroying nearby tissues in the process. This causes inflammation, which in turn causes the production of cytokines. Tumor biomarkers may be able to be utilized to correlate the clinical course of OSCC, according to mounting evidence [1].

The markers associated with blood tumors can be divided into two groups: those associated with immune responses and those associated with tumors that are secreted or released from the tumor. Squamous cell carcinoma antigen (SCC-Ag), cytokeratin, and the recently discovered molecular markers were among the tumor-related markers found in OSCCs. Tumor necrosis factor, interleukins, C-reactive protein (CRP), and other markers are associated with immune responses [4].

Within hours of tissue damage, the liver produces CRP, an acute-phase infectious and inflammatory process protein that can indicate an infection or an inflammatory state. CRP is released into the bloodstream [5].

A strong correlation was found between elevated CRP and bony erosion, extracapsular spread, nodal positivity, and advanced stage. These findings suggest that elevated CRP functions as a marker for chronic inflammation within the tumor microenvironment, which in turn stimulates angiogenesis and cell proliferation and inhibits apoptosis [6].

This study sought to determine the significance of CRP as a tumor marker in patients with OSCC at the time of initial diagnosis in relation to lymph node metastasis, age, gender, tumor size, and histologic grading.

## Materials and methods

Patients reporting to the outpatient department of oral and maxillofacial surgery at Bharati Vidyapeeth Dental College and Hospital, Pune, India, were included in this study. The study comprised 53 participants divided into malignant and pre-malignant groups. The malignant group included 39 participants with OSCC whereas the pre-malignant group comprised 14 patients that included patients with leukoplakia, erythroplakia, oral submucous fibrosis, and lichen planus.

Patients suffering from systemic conditions such as cardiovascular diseases, anemia, liver diseases, kidney diseases, blood dyscrasias, history of stroke or any autoimmune diseases, patients under 20 years of age, patients undergoing chemotherapy or radiotherapy, and patients with recurrence of lesions were excluded from the study. An informed consent was procured after explaining the aim of the study to the patients.

Before performing a biopsy of the suspected lesions, the patient will be sent for routine blood and urine investigations, along with which, a CRP test will also be done. The collected blood was subjected to centrifugation to separate the serum. CRP levels were estimated using immunoturbidimetry which is an in-vitro diagnostic assay from quantitative determination of CRP in human serum and plasma.

The consent was obtained or waived by all participants in this study and the Institutional Ethics Committee of Bharati Vidyapeeth (Deemed to be University) Dental College and Hospital, Pune issued approval (EC/NEW/INST/2021/MH/0029) for the study.

Data was entered in an Excel sheet (Microsoft Corporation, Redmond, United States). Descriptive statistics of the study participants were expressed as means and standard deviation, frequencies, and percentages. The CRP levels were categorized as negative if values were <5 mg/L and positive if values were ≥5 mg/L. The association of CRP levels with the different clinicopathological features was done using the chi-square test. The correlation of CRP value with pre-malignant and malignant lesions was done using Pearson's correlation test. A p-value less than or equal to 0.05 was considered to be statistically significant. All analyses were performed using IBM SPSS Statistics for Windows, Version 25 (Released 2017; IBM Corp., Armonk, New York, United States).

## Results

Malignancy status distribution of the study participants

About 53 participants (N=53) in total participated in the study; of these, 39 belonged to the malignant group (N=39, 73.58%) and 14 to the pre-malignant group (N=14, 26.42%) as described in Table 1. This study involved a number of malignant cases as demonstrated by Table 1.

**Table 1:** Malignancy status of the study participants The data has been represented as N, %.

Malignancy status	Number	Percentage
Pre-malignant cases	14	26.42%
Malignant cases	39	73.58%
Total	53	100%

Age and gender distribution of the study participants

The study included a total of 53 participants (N=53), out of which the malignant group consisted of 28 male participants (N=28, 71.8) and 11 female participants (N=11, 28.2%) whereas the pre-malignant group consisted of 7 male participants (N=7, 50%) as well as 7 female participants (N=7, 50%) which is denoted in Table 2. The mean age of the malignant group was 54.5±11.05 and the mean age of the pre-malignant group was 45.43±15.12. Thereby, Table 2 describes the population group involved with the pre-malignant and malignant lesions are mostly individuals in their fourth or fifth decade of life.

**Table 2:** Age distribution of the study participants The data has been represented as mean±SD.

Age (in years)	Mean	Standard deviation
Pre-malignant group (N=14)	45.43	15.12
Malignant group (N=39)	54.59	11.05
Total (N=53)	52.17	12.77

Site distribution of the study participants

Malignant Participants

The malignant lesions were observed in six different sites of the oral cavity. The most commonly involved site was the tongue (N=11, 28.2%) and the buccal mucosa (N=10, 25.6%), respectively. This was followed by alveolus (N=7, 17.9%), retromolar trigone (N=6, 15.4%), the floor of the mouth (N=3, 7.7%), and lip (N=2, 5.1%) which is represented in Table 3. The following table describes the variations in the site of the lesions.

**Table 3:** Site of primary cancer of the malignant study participants The data has been represented as N, %.

Site of primary cancer	Number	Percentage
Alveolus	7	17.9%
Buccal mucosa	10	25.6%
Floor of mouth	3	7.7%
Lip	2	5.1%
Retromolar trigone	6	15.4%
Tongue	11	28.2%
Total	39	100%

Pre-malignant Participants

The lesions in the oral cavity that were pre-malignant were limited to three different sites. These lesions were most commonly observed in the buccal mucosa (N=9, 64.3%), followed by the tongue (N=3, 21.4%) and the retromolar trigone (N=2, 14.3%), described in Table 4. The following table describes the variation in the site of the lesions in patients participating in the study.

**Table 4:** Site of pre-malignancy of the study participants The data has been represented as N, %.

Site of primary cancer	Number	Percentage
Buccal mucosa	9	64.3%
Retromolar trigone	2	14.3%
Tongue	3	21.4%
Total	14	100%

Mean CRP values of the study participants

The mean CRP value was calculated for all the study participants. The CRP levels were categorized as negative if values were <5 mg/L and positive if values were ≥5 mg/L. The malignant group had a mean CRP of 7.90±3.18 and the pre-malignant group had a mean of 2.46±1.79. The total CRP mean was 6.46±3.75. The values are significantly mentioned in the Table 5. 

**Table 5:** Mean CRP values of the study participants The data has been represented as mean±SD.

CRP value (mg/L)	Mean	Standard deviation
Pre-malignant group (N=14)	2.46	1.79
Malignant group (N=39)	7.90	3.18
Total (N=53)	6.46	3.75

Correlation of CRP with the malignancy status

The correlation of CRP value with pre-malignant and malignant lesions was done using Pearson's correlation test where a p-value ≤ 0.05 was considered to be statistically significant. The correlation coefficient was 0.645 and the Pearson's correlation test value was <0.001. Thus, the p-value had a strong statistical relationship. The test significance is denoted by Table 6.

**Table 6:** Correlation of CRP with the malignancy status *p-value ≤ 0.05 is statistically significant.

Correlation coefficient	p-value (Pearson's correlation test)
0.645	<0.001*

## Discussion

OSCC is one of the most common cancers of the oral cavity which is a major global health concern with a low five-year survival rate [7,8]. The majority of cancers start as benign precancerous lesions, out of which only a small percentage of them go on to become malignant. Studies have found few histopathological factors that contribute to the precancerous lesion's malignant transformation including immunological dysregulation, an increase in the mutation burden, particular alterations to signaling pathways, and epidermal growth factor receptor amplification [9-11].

There is a direct correlation between cancer and inflammation. According to certain theories, inflammatory cells function as early intrinsic defense mechanisms in malignant tumors to withstand the tumor. However, through tumor angiogenesis and DNA damage, persistent inflammation brought on by infections and chemical irritants is crucial to the development of cancer [12,13]. Neutrophil count, lymphocyte count, CRP, albumin (alb), neutrophil-lymphocyte ratio (NLR), platelet lymphocyte ratio (PLR), modified Glasgow Prognostic Score (mGPS), and prognostic nutritional index (PNI) are among the serological tests linked to inflammation [14,15]. When elevated CRP levels are found, OSCC patients with larger tumors and more advanced tumor stages have a lower overall survival rate; thus, elevated CRP levels can be considered a prognostic parameter [16,17].

After tissue damage, the liver produces CRP, an acute phase protein that can indicate infection or inflammation. CRP is released into the bloodstream a few hours later. Increased CRP level before surgery is linked to larger tumors, more advanced stages, and a lower overall survival rate in OSCC patients.

Elevated CRP levels could be indicative of a degree of skin or bone invasion, tumor status, and lymph node metastasis. In addition, it can be used to prevent and identify precancerous lesions early on, which will increase the prognosis for individuals who develop oral cancer [18,19].

Traditional biopsy has always been considered the gold standard for the diagnosis of oral pre-malignant and malignant lesions. However, various instances have been noted wherein a false-positive or a false-negative biopsy report has been encountered. This misdiagnosis results in unwarranted aggressive surgical intervention.

For the study, the CRP levels were categorized as positive if they were greater than 5 mg/L and negative if they were less than 5 mg/L. There was a finding of a marked increase in CRP levels in malignant as compared to pre-malignant lesions. Furthermore, CRP levels reveal an upward trend with the TNM stages. Also, we encountered all positive CRP cases in stages II, III, and IVa thereby acting as a potential indicator for malignancy. According to various other studies, CRP, thus, can be considered a liquid biopsy and can be utilized as an adjunct along with a traditional biopsy.

According to the results of George et al., pre-malignant lesions typically affect individuals between the ages of 50 and 69. The age range of the patients in our study with pre-malignant lesions was 20 to 75 years, with a mean age of 45.43 years. Numerous etiological factors, including alcohol, tobacco, infections, genetics, immunosuppression, and malnutrition, could be to blame for this. The majority of the malignant patients in our study were older, with a mean age of 54.59 years, spanning from 40 to 76 years of age. Radhakrishnan et al. state that oral cancers usually affect people in their fifth and sixth decades of life [20,21].

Seven male patients (50%) and seven female patients (50%) in our study had pre-malignant lesions. George et al. claim that pre-malignant lesions are more common in men because of increased tobacco and alcohol use as a habit. However, our study's results showed an equal number of male and female patients, which is inconsistent with their findings and may have to do with the patient's demographics [20]. The malignant lesions were observed in 39 patients which included 28 male patients (71.8%) and 11 female patients (28.2%). According to Watanabe et al., oral cancer is generally more prevalent in males but does vary according to the demographics which is in accordance with our study. However, another study conducted by Tariq et al. reported a male-to-female ratio of 1:1.6 thereby reporting a higher number of female patients which contradicts our study [22,23].

All of the patients in our study had SCC diagnosed after a histopathological examination. About 39 patients (73.58%) out of the 53 participants in total received an OSCC diagnosis. Viviano et al. state that there exists a close correlation between the incidence of oral carcinoma and specific customs and habits of various populations, including alcohol consumption and smoking. Our study included patients who were tobacco users both in chewable and smoking forms with a combination of tobacco and quid being most common [24]. According to our study, there were equally many patients with pre-malignant lesions that may have been brought on by alcohol or tobacco use. Parlatescu et al. state that smoking is frequently linked to leukoplakia, a lesion that is far more common in smokers than in non-smokers [25].

Pre-malignant lesions were most frequently found in the buccal mucosa in our study, then the tongue and the retromolar trigone. Neville and Day reported that the lower lip, alveolar mucosa, and buccal mucosa were the most frequently observed sites for pre-malignant lesions. Because these areas were in constant contact with the etiological agents, like tobacco, they were most frequently affected [26]. The tongue was the most frequently found site of malignancy in the current study, followed by the buccal mucosa, alveolus, and retromolar trigone. The tongue is a common site of OSCC, followed by the retromolar trigone, buccal mucosa, floor of the mouth, hard palate, and gingivae, according to Viviano et al. This is in line with the findings of Radhakrishnan et al., who showed that the location of betel quid usually corresponds with the site of origin of oral cancer [24,21].

In comparison to patients with pre-malignant lesions, patients with OSCC had significantly higher CRP concentrations, according to our investigation. The mean CRP level in pre-malignant lesions was 2.46±1.79 and in malignant patients was 7.90±3.18; there was a statistically significant difference between the two groups (p<0.001). These outcomes were in line with those of a study by Metgud and Bajaj, which found that patients with malignant lesions had mean CRP levels that were higher than those of patients without lesions, both normal and pre-malignant [27]. CRP is considered to be elevated in our study when its value is greater than 5 mg/L, which is highly statistically significant adding to the prognostic value.

The malignant group in the current study had CRP values with a mean±SD of 7.90±3.18. Higher TNM staging was shown to be correlated with higher CRP levels in a related study by Tariq et al. The positive CRP values that match the TNM stages serve as evidence for this. Every TNM stage II, III, and IVa that the study patients experienced had a positive CRP value (≥5 mg/L). According to the findings of the Tariq et al. study, patients with higher pre-operative serum CRP levels have poorer stages and, consequently, lower survival rates than patients with lesser stages; as a result, elevated pre-operative CRP levels are predictive markers in patients with OSCC [22].

It is a quick, repeatable, and reasonably priced diagnostic method. As a result, CRP has the potential to be clinically relevant in assisting with patient identification, prognosticating the course of the disease, and serving as an adjuvant for existing laboratory biomarkers in pre-malignant and malignant lesions.

CRP-level findings can also be utilized for operated malignancy patients' follow-up regarding recurrence or in pre-malignant cases that might have undergone malignant transformation.

According to the current study, patients with malignant lesions had higher serum CRP levels than patients with pre-malignant lesions. This finding raises the possibility that CRP is a biomarker that can be used to gauge the severity of a disease. Serum CRP levels that are elevated are linked to a poor prognosis, so CRP can help manage these conditions. It is still unknown, though, whether elevated CRP levels occur prior to the biological onset of cancer or whether they serve as a risk factor for the onset of cancer. Therefore, more research is needed to assess serum CRP levels before and after treatment in larger sample sizes to ascertain the disease status.

## Conclusions

The development, spread, and metastasis of cancer are all significantly influenced by chronic inflammation. CRP is a marker utilized by clinicians for indicators of inflammation and systemic illnesses. According to the current study, patients with malignancy had higher serum CRP levels than patients with pre-malignant lesions, indicating that CRP is a biomarker that can be used to gauge probable malignant transformation and advise biopsy immediately. Serum CRP levels that are elevated are linked to a poor prognosis, invasiveness, and higher-stage disease.

In our study, 5 mg/L of CRP value was statistically significant denoting the presence of malignancy, so, we recommend recording CRP values as routine investigations for pre-malignant conditions as a guideline for performing biopsy for early detection of malignancy. Similarly, these findings will help in assessing the prognostic value before surgery and act as an adjuvant for confirmation of histopathological reports, whenever the clinician is in doubt regarding the presence or absence of malignant transformation. Within the limits of this study, CRP can be used only as an adjuvant to assess the histopathological reports in cases of doubt. Therefore, more research is needed to assess serum CRP levels before and after treatment in a larger sample.

## References

[1] Singhavi HR, Khare N, Singh A (2021). Impact of pre-operative serum C-reactive protein and cell-free chromatin levels on tumor aggressiveness and survival outcome in oral cavity squamous cell carcinoma. Oral Oncol.

[2] Park HC, Kim MY, Kim CH (2016). C-reactive protein/albumin ratio as prognostic score in oral squamous cell carcinoma. J Korean Assoc Oral Maxillofac Surg.

[3] Sarode G, Maniyar N, Sarode SC, Jafer M, Patil S, Awan KH (2020). Epidemiologic aspects of oral cancer. Dis Mon.

[4] DE Paz D, Young CK, Chien HT (2019). Prognostic roles of SCC antigen, CRP and CYFRA 21-1 in oral cavity squamous cell carcinoma. Anticancer Res.

[5] Tai SF, Chien HT, Young CK (2017). Roles of preoperative C-reactive protein are more relevant in buccal cancer than other subsites. World J Surg Oncol.

[6] Huang SF, Wei FC, Liao CT (2012). Risk stratification in oral cavity squamous cell carcinoma by preoperative CRP and SCC antigen levels. Ann Surg Oncol.

[7] Jemal A, Bray F, Center MM, Ferlay J, Ward E, Forman D (2011). Global cancer statistics. CA Cancer J Clin.

[8] Küffer R, Lombardi T (2002). Premalignant lesions of the oral mucosa. A discussion about the place of oral intraepithelial neoplasia (OIN). Oral Oncol.

[9] Foy JP, Bertolus C, Ortiz-Cuaran S (2018). Immunological and classical subtypes of oral premalignant lesions. Oncoimmunology.

[10] Farah CS, Jessri M, Bennett NC, Dalley AJ, Shearston KD, Fox SA (2019). Exome sequencing of oral leukoplakia and oral squamous cell carcinoma implicates DNA damage repair gene defects in malignant transformation. Oral Oncol.

[11] Das D, Maitra A, Panda CK, Ghose S, Roy B, Sarin R, Majumder PP (2021). Genes and pathways monotonically dysregulated during progression from normal through leukoplakia to gingivo-buccal oral cancer. NPJ Genom Med.

[12] Hussain SP, Harris CC (2007). Inflammation and cancer: an ancient link with novel potentials. Int J Cancer.

[13] van Kempen LC, de Visser KE, Coussens LM (2006). Inflammation, proteases and cancer. Eur J Cancer.

[14] Khandavilli SD, Ceallaigh PO, Lloyd CJ, Whitaker R (2009). Serum C-reactive protein as a prognostic indicator in patients with oral squamous cell carcinoma. Oral Oncol.

[15] Jin Y, Zhao L, Peng F (2013). Prognostic impact of serum albumin levels on the recurrence of stage I non-small cell lung cancer. Clinics (Sao Paulo).

[16] Singh N, Baby D, Rajguru JP, Patil PB, Thakkannavar SS, Pujari VB (2019). Inflammation and cancer. Ann Afr Med.

[17] Jeng JH, Wang YJ, Chiang BL (2003). Roles of keratinocyte inflammation in oral cancer: regulating the prostaglandin E2, interleukin-6 and TNF-alpha production of oral epithelial cells by areca nut extract and arecoline. Carcinogenesis.

[18] Nozoe T, Saeki H, Sugimachi K (2001). Significance of preoperative elevation of serum C-reactive protein as an indicator of prognosis in esophageal carcinoma. Am J Surg.

[19] Gockel I, Dirksen K, Messow CM, Junginger T (2006). Significance of preoperative C-reactive protein as a parameter of the perioperative course and long-term prognosis in squamous cell carcinoma and adenocarcinoma of the oesophagus. World J Gastroenterol.

[20] George A, Sreenivasan BS, Sunil S (2011). Potentially malignant disorders of oral cavity. Oral Maxillofac Pathol J.

[21] Radhakrishnan R, Shrestha B, Bajracharya D (2012). Oral Cancer - An Overview. https://www.researchgate.net/publication/221928239_Oral_Cancer_-_An_Overview.

[22] Tariq FA, Janjua OS, Khan U (2011). C-reactive protein as a prognostic indicator of oral squamous cell carcinoma - a retrospective study. Pak Oral Dent J.

[23] Watanabe N, Ohkubo T, Shimizu M, Tanaka T Preneoplasia and carcinogenesis of the oral cavity. Oncol Discov.

[24] Viviano M, Addamo A, Lorenzini G (2013). Oral cancer. Int J Clin Dent.

[25] Parlatescu I, Gheorghe C, Coculescu E, Tovaru S (2014). Oral leukoplakia - an update. Maedica (Bucur).

[26] Neville BW, Day TA (2002). Oral cancer and precancerous lesions. CA Cancer J Clin.

[27] Metgud R, Bajaj S (2016). Altered serum and salivary C-reactive protein levels in patients with oral premalignant lesions and oral squamous cell carcinoma. Biotech Histochem.

